# Fertility, Gestation Outcome and Parasite Congenital Transmissibility in Mice Infected with TcI, TcII and TcVI Genotypes of *Trypanosoma cruzi*

**DOI:** 10.1371/journal.pntd.0002271

**Published:** 2013-06-13

**Authors:** Sabrina Cencig, Nicolas Coltel, Carine Truyens, Yves Carlier

**Affiliations:** Laboratoire de Parasitologie, Faculté de Médecine, Université Libre de Bruxelles (ULB), Brussels, Belgium; Harvard School of Public Health, United States of America

## Abstract

This work aims to compare the effects of acute or chronic infections with the *T. cruzi* genotypes TcI (X10 strain), TcII (Y strain) and TcVI (Tulahuen strain) on fertility, gestation, pup growth and the possible vertical transmission of parasites in BALB/c mice. The occurrence of congenital infection was evaluated by microscopic examination of blood and/or qPCR on blood and heart in newborn pups and/or older offspring submitted to cyclophosphamide-induced immunosuppression in order to detect possible cryptic congenital infection. Altogether, the results show that: i) for the three strains tested, acute infection occurring after the embryo implantation in the uterus (parasite inoculation 4 days before mating), or close to delivery (parasite inoculation on day 13 of gestation), prevents or severely jeopardizes gestation outcome (inducing pup mortality and intra-uterine growth retardation); ii) for the three strains tested, gestation during chronic infection results in intra-uterine growth retardation, whereas re-inoculation of TcVI parasites during gestation in such chronically infected mice, in addition, strongly increases pup mortality; iii) congenital infection remains a rare consequence of infection (occurring in approximately 4% of living pups born to acutely infected dams); iv) PCR, detecting parasitic DNA and not living parasites, is not convenient to detect congenial infection close to delivery; v) transmission of parasites by breast milk is unlikely. This study should encourage further investigations using other parasite strains and genotypes to explore the role of virulence and other factors, as well as the mechanisms of such effects on gestation and on the establishment of congenital infection.

## Author Summary

The association between the infection with *T. cruzi*, the agent of Chagas disease (a neglected infectious disease), and pregnancy is frequent in Latin American endemic areas and occurs also in non-endemic areas. Information on the relationship between parasite genotypes (differently distributed in the endemic areas) and their effect on pregnancy is scarce. *T. cruzi* parasites are heterogeneous complexes of genetic lineages presently divided into six main genotypes (TcI to TcVI). Experimental studies might bring information on the effects of *T. cruzi* genotypes on gestation and on their potential role in congenital transmission and infection. The present work compares the effects of acute or chronic infections with three *T. cruzi* strains, belonging to the genotypes TcI, TcII and TcVI, on gestation outcome and the possible vertical transmission of parasites in mice. For the three strains tested, we show that acute infection, occurring during gestation, severely jeopardizes its outcome, whereas gestation during chronic infection mainly results in intra-uterine growth retardation. Moreover, we also show that congenital infection remains a rare consequence of dam infection and that transmission of parasites by breast milk is unlikely.

## Introduction

Chagas disease, caused by the kinetoplastid flagellate *T. cruzi*, is one of the major causes of cardiac failure in Latin America. This trypanosomiasis has become a global public health problem due to migrations of Latin Americans to non-endemic countries, particularly to United States, Europe, Japan and Australia. This parasite, infecting 8 to 10 million people, can be transmitted by vector bugs, orally, by transfusion of infected blood or organ transplantations as well as from mother-to-child [1], [2]. Owing to the successful implementation in Latin America of national programs aiming to control home vector infestation and blood transfusion, the relative importance of congenital transmission has recently increased [3].

Maternal-fetal transmission occurs in endemic as well as non-endemic areas and from one generation to another, allowing spread of parasite infection for long periods of time. At least 2 million women of the fertile age are estimated to be infected with *T. cruzi* in Latin America. Congenital transmission occurs in up to 12% of pregnant and chronically infected women (average around 4–6%) with an estimated number of congenitally infected newborns >15 000 per year [3], [4]. The incidence of congenital cases in non-endemic areas is not known, although several reports attest to its occurrence [5]–[7]. Contradictory data have been reported on the frequency of abortions, stillbirths, premature births and low birth weight occurring in chronically infected versus uninfected mothers living in the same areas [8]–[12], whereas no significant effects of maternal chronic infection have been reported on growth of uninfected fetuses/neonates born to infected mothers [13].

*T. cruzi* parasites are heterogeneous complexes of genetic lineages presently divided in six main genotypes (TcI to TcVI; reviewed in [14]). All *T. cruzi* genotypes, with the exception of TcIV, have been identified in human cases of congenital Chagas disease. The TcV genotype has been reported in most of congenital cases in Argentina, Bolivia, Southern Brazil, Chile and Paraguay, whereas the other genotypes have been identified more sporadically [15]–[21]. The distribution of genotypes in these congenital cases being similar to that observed in the local infected population [16], [17], [19], there is no clear evidence of a relationship between *T. cruzi* genotypes and an eventual tropism for congenital transmission and infection in human fetuses. Moreover, no information is available on the effect of the different *T. cruzi* genotypes on pregnancy.

Experimental studies might bring information on the potential role of *T. cruzi* genotypes on gestation and congenital transmission. We, along with others reported that TcVI infection just before mating strongly reduced mouse fertility [22], [23], whereas previous studies did not observed any effect [24], [25]. TcVI, as well as TcI, TcII or other strains of undefined genotypes, seem to induce fetal growth retardation when inoculated during gestation [26]–[28] or when gestation occurs in chronic infection [29]. Maternal-fetal transmission of parasites was not observed or rarely observed (by investigation of blood parasites, hemoculture or histological studies in offspring) in mice or rats inoculated with various *T. cruzi* strains (belonging to TcI, TcII, TcVI or undefined genotypes), either a long time [27], [29]–[31], or just before or during gestation [23], [25], [26], [30], [32]–[34]. Such congenital transmission seems independent of the placental parasite invasion [22], [33], [35]. Higher transmissibility rates could be obtained when placental lesions or blockade of placental phagocytic activity were induced [36], [37]. By contrast, two other studies reported 33% to 66% positive PCR in offspring of mice chronically infected with TcI , TcIV or TcV [38], [39], but congenital infection was not demonstrated in such pups.

The present work aims to compare the effects of acute and chronic infections with the *T. cruzi* genotypes TcI, TcII and TcVI on fertility, gestation, pup growth and the possible vertical transmission of parasites, in BALB/c mice. The occurrence of congenital infection was evaluated by microscopic examination of blood and/or qPCR on blood and heart, in newborn pups and/or older offspring submitted to cyclophosphamide-induced immunosuppression in order to detect possible cryptic congenital infection.

## Materials and Methods

### *T. cruzi* genotypes

The used parasites, belonging to the TcI, TcII and TcVI genotypes, are reference strains for laboratory investigations: X10, Y and Tulahuen, respectively [14]. The latter, coming from human cases of Chagas disease, have been previously cloned and their genotype was checked regularly. Tc II and TcVI strains were stabilised since years in our laboratory, in immune competent BALB/c mice in which significant parasitemia could be obtained [29], [40]. The used TcI strain was kindly given by M. Miles and M Lewis (London School of Tropical Medicine and Hygiene, LSTMH, London, UK) as a culture of epimastigotes with a low amount of metacyclic trypomastigotes, unsuitable for direct mouse infection. The parasite mixture was cultured in a Grace modified metacyclogenic medium [41] in order to increase its proportion of metacyclic trypomastigotes. Collected parasites were used to infect mice previously submitted to immune depression with cyclophosphamide (CP) (see below). The latter were inoculated subcutaneously (*s.c*) in the back with at least 10^6^ parasites per mouse. Blood trypomastigotes, collected from such mice at the moment of the highest parasitemias, were serially passed to other animals. After multiple consecutive passages, parasites were transferred to immune-competent BALB/c mice in order to obtain stabilized and reproducible infections suitable for gestation studies.

### Mice, ethics statement and determination of parasite blood levels

Experiments were performed with BALB/c mice (8 weeks old), purchased from Janvier (Le Genest-St-Isle, France). Animals were housed in our accredited animal facility in compliance with the guidelines of the “Université Libre de Bruxelles” (ULB) Ethic Committee for the use of laboratory animals, adhering to the Belgian legislation on protection of such animals (protocol 51 approved by CEBEA, Brussels, Belgium).

Parasitemias in dams and pups were determined either by microscopic examination of tail vein blood, with a detection limit of 10,000 parasites/mL as previously described [42], or estimated by qPCR analysis (see below) when blood microscopic examinations were negative.

### Evaluation of mouse reproductive capacity, gestation and pup growth

In order to evaluate mouse reproductive capacity, females (bred in the absence of males) were placed first for 2 days in cages having contained males to synchronize their estrous cycles, as described earlier [22]. Mating was then performed by adding one male for 2 females for 5 days. Females were checked each day for the presence of a vaginal plug (VP). The day when VP occurred was considered as the day 0 of gestation (G0). Each identified VP positive female was set apart in a new cage. Their weights were recorded at G0 and G10. VP+ mice having gained weight at G10 (±3 g from G0) were considered gravid and weights were further monitored at G13, G15, G17 and G19. Litter sizes and pup mortality rates were recorded at delivery. Pups were weighted weekly from birth to the end of the experiments.

### Mouse groups and infection of animals

**Figure 1:**
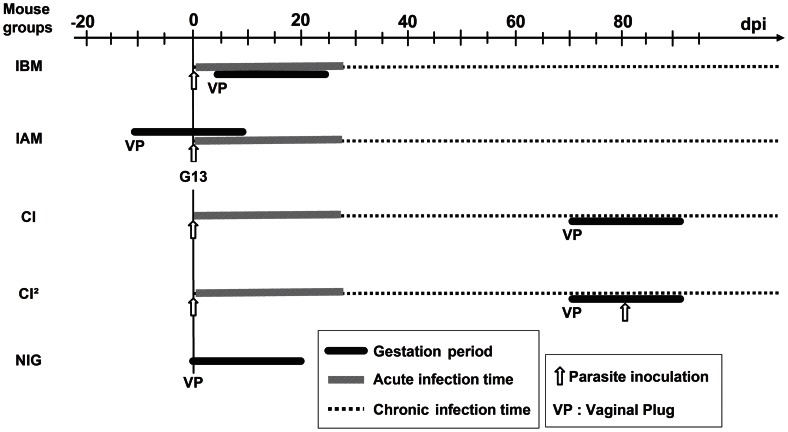
*T.* cruzi infection *versus* gestation timing in the mouse groups. IBM = female mice acutely infected 4 days before mating; IAM = female mice acutely infected after mating (on G13); CI = female mice mated during the chronic infection (on dpi74); CI^2^ = female mice mated during the chronic infection and re-infected on G8–G10; NIG = non-infected and gravid mice.

As schematized in Fig. 1, the following groups of female mice were considered for evaluating the effect of TcI, TcII and TcVI infection on reproductive capacity, gestation and/or vertical transmission of parasites: i) inoculated with parasites 4 days before mating (IBM group for Infection Before Mating), so that the ascending phase of parasitaemia ran primarily soon after the embryo implantation (occurring at G4.5–6 [43], [44]; ii) inoculated on G13, i.e. after mating (IAM group for Infection After Mating), so that the ascending phase of parasitaemia occurred at delivery (around G19-G21); iii) mated on day post-infection (dpi) 74, i.e. during the chronic infection (CI group); iv) mated during the chronic infection (dpi 74) and re-inoculated on G8–G10 (CI^2^ group); v) mated and non-infected (NIG group for Non-Infected and Gravid). The IBM, IAM and CI groups were also compared to their respective infected and non-gravid animal groups (ING for Infected and Non-Gravid) to evaluate the effect of gestation on the course of the infection.

Preliminary experiments were performed in order to define the inoculum sizes for each *T. cruzi* genotype to be used to avoid the mortality of animals during the period of the experiments, as well as to obtain infection kinetics required for the animal groups defined above. The following inoculum sizes were used according to the mouse group: IBM: 10^6^ TcI or 10^3^ TcII; IAM: 10^5^ to 10^6^ TcI, TcII, or TcVI; CI: 10^6^ TcI or 10^3^ TcII/TcVI; CI^2^ (reinfection): 10^2^ TcVI. Since our previous works showed that similar infection kinetics were obtained by *ip* and *sc* inoculation routes [22], and in order to avoid direct interaction with the reproduction process, parasites were inoculated subcutaneously either in the back (IBM and IAM) or in the foot pad (CI^2^), whereas CI mice were infected by the *ip* route. Details of inoculum sizes and numbers of used mice per group are indicated in Table 1.

**Table 1:** Effect of infection with TcI, TcII and TcVI on reproductive capacity of BALB/c mice.

MG	PG	PI	N_1_	Mat. rate^a^ %	Gest. rate^b^ %	Litter size m ± SEM (N_2_)	Pup mortality^c^ n/N_3_ (%)	Pup weight (g) m ± SEM
**NIG**	**-**	**-**	205	88.8	48.2	5.7±0.3 (87)	28/500 (5.6)	1.48±0.01
**IBM**	**TcI**	10^6^	16	81.3	69.2	0.0^d^ 6.5±2.5 (2)	**-** 4/13 (30.8)	**-** 1.20± 0.05***
	**TcII**	10^3^	19	84.2	68.7	0.0^d^	-	-
**IAM**	**TcI**	10^6^	41	84.0	38.1	4.4±0.4 (13)	8/58 (13.8)*	1.40±0.02*
	**TcII**	10^5^	61	86.4	64.0	5.9±0.3 (34)	21/200 (10.5)*	1.41±0.01***
	**TcVI**	10^5^	25	95.4	51.6	6.4±0.7 (12)	7/76 (9.2)	1.25±0.05**
	**TcVI**	10^6^	32	96.0	41.7	5.4±0.4 (13)	6/70 (8.6)	1.33±0.03**
**CI**	**TcI**	10^6^	10	80.0	50.0	8.0±1.0 (4)	0/32 (0.0)	1.26±0.05*
	**TcII**	10^3^	15	93.3	42.8	5.0±1.3 (6)	2/30 (6.7)	1.40±0.02*
	**TcVI**	10^3^	35	96.4	48.1	5.2±0.4 (16)	10/84 (11.9)*	1.33±0.02**
**CI^2^**	**TcVI**	10^3^/10^2^	39	100.0	38.5	6.3±0.6 (15)	25/95 (26.3)*	1.23±0.04**

MG = mouse group (see Figure 1 for group nomenclature); PG = parasite genotype; PI = parasite inoculum; N_1_ = number of studied dams; Mat. Rate = mating rate; Gest. Rate = gestation rate; N_2_ = number of females that delivered pups; N_3_ = number of delivered pups; n = number of dead pups;

a = determined by the occurrence of vaginal plug;

b = determined by weight increase at G10 among females displaying a positive vaginal plug ;

c = on the delivery day;

d = termination of gestation at G12;

* = P<0.05,

** = P<0.01,

*** = P<0.001, by comparison to the NIG group.

### Cyclophosphamide-induced immune suppression in mice

In order to reactivate eventual cryptic infection, pup or adult mice received 3 or 4 *i.p.* injections of 200 mg/kg of CP (Endoxan, Baxter, Lessines, Belgium), respectively, on alternate days, as previously described [45], [46]. The efficacy of such immune suppression procedure to assess cryptic infection was verified by the high parasitemias (microscopic examination) and mortality rates of 100% of mice chronically infected with TcVI and having received CP.

### Evaluation of vertical transmission of parasites

Parasites can theoretically be transmitted through the placenta, leading to congenital infection at birth, or after birth by breastfeeding (from birth to the weaning time) [3], [47]. In order to evaluate both possibilities, some pups from IAM mice were nursed by NIG lactating dams, and offspring from NIG healthy dams was suckled by IAM females (cross-fostering performed at birth). This procedure also allowed observing pup growth in normal suckling conditions, since acutely infected dams (IBM and IAM) might deliver nursing insufficient enough to their growth.

Some pups of infected dams were sacrificed by gaseous anesthesia at birth (D0) and DNA extracts (see below) of whole pups were submitted to qPCR analyses. Others were sacrificed on D15 or D35 to collect blood (examined by microscopy or qPCR) and heart (qPCR). Finally, other pups were left alive to investigate their antibody levels by ELISA (plasma) and to be submitted to CP-immune depression on D30–35 (see above), sacrificed on D45–50 and studied as mentioned above for D15 pups.

### DNA extraction, generation of tissue standards and Real-Time quantitative PCR

Blood, heart and entire pups DNA extraction, as well as generation of blood and heart standards (from tissues collected on uninfected mice and spiked with parasites) were performed as previously described [16], [46], [48].

Real-time qPCR used the *T. cruzi-*specific TcZ1 and TcZ2 primers or GAPDH primers in a LightCycler 480 system (Roche Diagnostics Brussels, Belgium) as previously described [46]. Each qPCR run contained 2 negative controls (no DNA added to the reaction), 2 positive controls (see below) and each DNA sample was quantified in duplicate and averaged before determining their parasite equivalent load by plotting their CP values against the tissue standards. The amount of tissue analyzed in each PCR reaction was normalized by dividing their TcZ DNA value by that of the murine GAPDH DNA (housekeeping gene) in the same sample.

Positive controls, such as BALB/c heart in acute or in chronic phases of TcVI infection displayed 6220±2170 and 65±43 equivalent parasites/50 ng tissue DNA, respectively. Results were considered negative in blood when <1 parasite DNA equivalent/mL, and as traces in tissues when <0.1 parasite DNA equivalent per 50 ng of total DNA.

### Determination of *T. cruzi*-specific antibody levels

Determination of antibody levels was performed by ELISA, as previously described [29], [49]. Briefly, mouse plasma samples 1∶50 diluted were incubated into wells of microtiter plates previously coated with 2.5 µg of *T. cruzi* trypomastigote soluble antigenic extract. Isotypes of binding antibodies were detected using rat anti-mouse IgG1 and IgG2a (Abcam, Cambridge, UK), followed by incubation with HRP-conjugated goat anti-rat IgG (Jackson Immunoresearch, West Grove, USA). Finally, TMB substrate (BD Biosciences, Erembodegem, Belgium) was added for colour development and absorbances (A) were read at 450 nm. The cut-off value was calculated as the mean of absorbances from NING mice +3 SD. A plasma sample of BALB/c mice chronically infected with TcVI was used as positive control. Antibody index values were calculated according the formula (A_sample_ - A_NING_)/(A_chronic_- A_NING_)×100. Since transmission of maternal IgG1 and IgG2a antibodies in mice (the main antibody isotypes synthetized during *T. cruzi* infection in mice) mainly occurs by breast-feeding [47], [49] and that pups born to infected dams were suckled by uninfected ones (see above), detection of *T. cruzi*-specific antibodies in offspring can be considered as a confirmation of congenital infection.

### Statistical analysis

Results were presented as means ± SEM. Comparisons of means between groups were performed using the Mann-Whitney *U*-test. Comparisons of proportions were carried out using chi-square test. All tests were performed using Graph Pad software (Prism 5 version 5.02).

## Results

### Effects of acute *T. cruzi* infection on mouse fertility and gestation outcome

As shown in Table 1, both TcI and TcII IBM mouse groups displayed mating and gestation rates similar to those of NIG control mice. However, the gestation-associated weight gain of most of them regularly declined from G11–G12 onwards compared to NIG animals (data not shown) and all (TcII) or most of dams (14/16, i.e. 87.5% for TcI) did not deliver pups. Moreover, the neonatal mortality rate of pups delivered by the 2 TcI-infected dams was significantly higher compared to NIG controls, and the 9 surviving pups displayed 20% lower birth weights than NIG pups. They, however, recovered normal weights after 7 days of nursing by NIG mice. The data concerning the effect of acute infection with TcVI before mating on mouse reproductive capacity have been previously reported [22].

When parasites were inoculated on G13, duration of gestation (data not shown) and mean litter sizes of all IAM groups (infected with TcI, TcII or TcVI) were similar to NIG controls. Pup mortality was significantly higher in the TcI and TcII infection groups than in NIG offspring. Inoculating 10^5^ or 10^6^ TcVI parasites in pregnant mice did not change significantly the studied parameters as compared with controls. All mean weights of surviving IAM pups at delivery were significantly lower than those of NIG animals (by 5 to 15%; p<0.001) (Table 1). As for IBM offspring, when suckled by NIG nursing mice, IAM pups recovered normal weights within 7 days (data not shown).

### Effects of chronic *T. cruzi* infection and reinfection on gestation outcome

For the three strains tested, chronic infection of female mice (CI) did not interfere with either the mating or the gestation rates, or the gestation durations (data not shown), or the mean litter sizes, compared to the control NIG group (Table 1). If pups of dams chronically infected with TcVI displayed a slightly higher mortality rate at delivery than those born to NIG mice, re-inoculation of TcVI parasites during chronic infection (CI^2^ group) induced a significantly higher pup mortality (26% vs 12%, respectively; p<0.05; Table 1). Live pups from all CI groups, as for IAM groups, also displayed lower mean birth weights than NIG animals (by 5 to 15%; P<0.05; Table 1) and recovered weights similar to controls, in as soon as 7 days after birth (when suckled by NIG dams; data not shown).

### Effect of gestation on *T. cruzi* infection course

**Figure 2:**
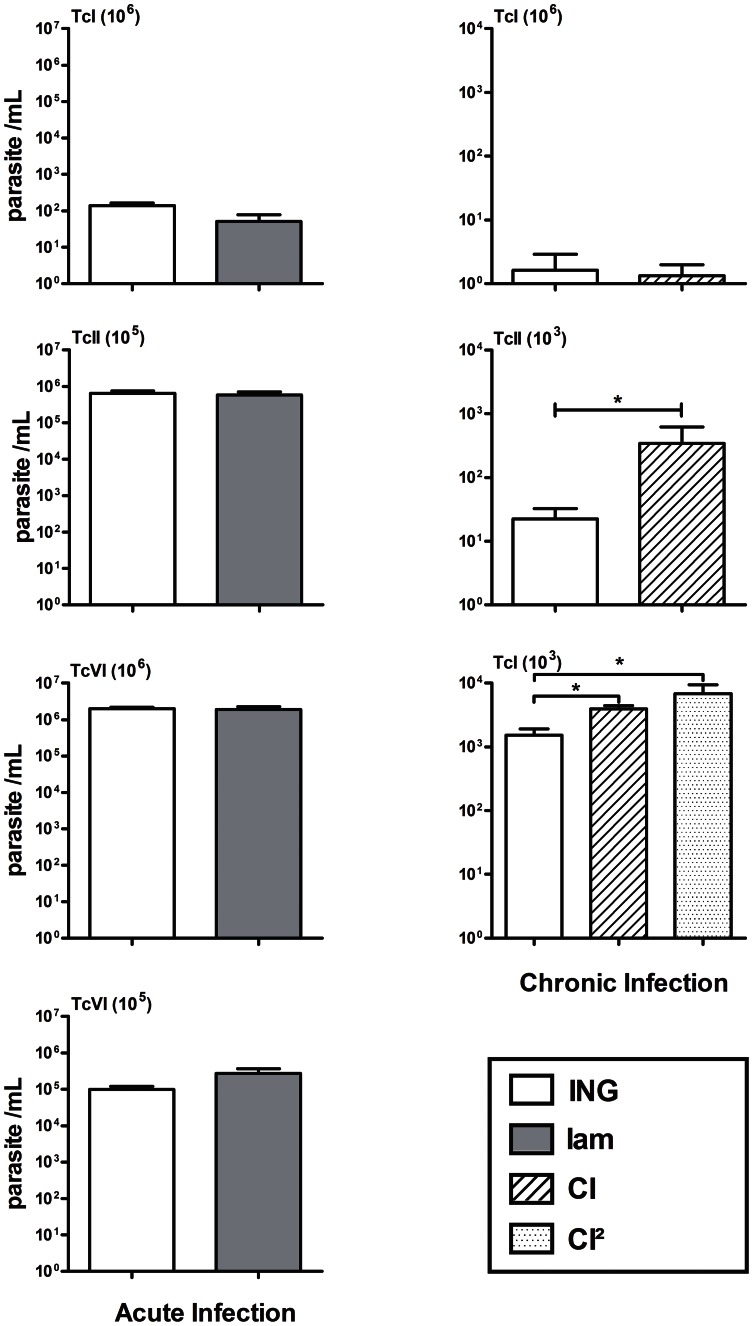
Parasitemias in dams acutely or chronically infected with TcI, TcII or TcVI. ING = infected and non-gravid mice; see Figure 1 for nomenclature of IAM, CI and CI^2^ groups; parasitemias were recorded at delivery for IAM (dpi 7–9), CI (dpi 94–96), CI^2^ dams (dpi 9–11 of reinfection) and at dpi 7 for ING mice; parasitemias in TcI acute-infection and TcII and TcVI chronic infections were recorded by qPCR, whereas those of acute TcII and TcVI infections were determined by blood microscopic investigation. * = P<0.05 by comparison to the ING group.

Mortality was not observed during the period of the experiment in infected mice either gravid or not. As shown in Fig. 2, mean parasitemias (determined at delivery by microscopic examination or qPCR) of IAM and ING dams, acutely or chronically infected with TcI, were much lower than in TcII- or TcVI-infected mice (0.05<P<0.001). If blood parasite levels of acutely infected IAM and ING dams were similar whatever the infecting *T. cruzi* genotypes, those of TcII and TcVI CI and CI^2^ groups were slightly boosted (by 2.5 to 15 fold) by comparison to their respective ING controls (P<0.05).

### Search for congenital infection

In order to detect congenital infection, a first set of experiments analyzed by qPCR pups born to mice infected with TcVI (inocumlum size: 10^5^). Positive qPCRs were observed in 8 to 62% of whole pups obtained at delivery (IAM suckled by NIG dams: n = 39; CI: n = 21; CI^2^: n = 47), and 16% (in blood) to 32% (in heart) of D15 pups (AIM: n = 31), whereas qPCR remained negative in D35 pups (IAM: n = 14; CI: n = 44; CI^2^: n = 43). However, the quantitative estimations of such positive qPCRs showed extremely low amounts or traces of DNA equivalent parasites As expected, all qPCR of pups from control NIG mice were negative (n = 10).

In order to verify if cryptic infection might be associated to such DNA traces detected close to the time of delivery, our further study protocol of congenital transmission (in IAM, CI and CI2 mouse groups infected with TcI, TcII and TcVI) was adapted as follows: i) microscopic examination of blood and investigation of antibodies in plasma of 15 and 30–35 days old offspring; ii) submission to CP-immune suppressive treatment (on D30–35) of all pups negative at previous microscopic examination, and iii) examination of blood (microscopy or qPCR) and/or heart (qPCR) of D45–50 immune suppressed pups. The following criteria were therefore defined *a priori* to validate offspring infection: i) detection of parasites in blood by direct microscopic examination before or after CP treatment, or, ii) detection of parasitic DNA in the blood or the heart (qPCR) after CP treatment on D45–50 after birth. Inversely, a negative qPCR result in the blood/heart in immune-suppressed offspring confirmed the absence of vertical transmission of parasites.

According to the criteria mentioned above, the 9 remaining alive pups of IBM mice and the 50 pups of IAM animals inoculated with 10^6^ TcI parasites did not show any congenital infection (Table 2). Experiments with higher TcI inoculum sizes were not feasible due to the weak amounts of blood parasites that we could obtain from infected mice (Fig. 2). By contrast, as reported in Table 2, congenital infection was detected in 6 among the 168 alive pups of IAM dams inoculated with 10^5^ TcII and in 2 out of the 50 alive pups of dams having received 10^6^ TcVI (i.e. a 3.6% and 4% congenital transmission rates, respectively). However, none of the 69 pups of females infected with 10^5^ TcVI displayed congenital infection. Congenital infection was no longer detected in offspring from CI mice infected with TcI, TcII and TcVI, or from CI^2^ mice infected with TcVI. The estimations of theoretical rates of congenital infection according to the numbers of studied pups per mouse group are indicated in Table 2.

**Table 2:** Congenital transmission of parasites in mice infected with TcI, TcII and TcVI.

Mouse group	Parasite genotype	Parasite inoculum	Congenital infection cases n/N		Congenital transmission rate %
			Blood microscopic examination	Blood/heart qPCR	
**IAM**	TcI	10^6^	0/50	0/50	0 (<2.0)
	TcII	10^5^	6/168	0/162	**3.6**
	TcVI	10^5^	0/69	0/69	0 (<1.4)
	TcVI	10^6^	2/50	0/48	**4.0**
**CI**	TcI	10^6^	0/32	0/32	0 (<3.1)
	TcII	10^3^	0/28	0/28	0 (<3.6)
	TcVI	10^3^	0/58	0/58	0 (<1.7)
**CI^2^**	TcVI	10^2^/10^9^	0/70	0/70	0 (<1.4)

See Figure 1 for group nomenclature; n = number of positive cases; N = total number of examined pups; the 8 congenitally-infected cases were detected by microscopic blood examination on D15 or D30 after birth; all the other examined pups remained negative at microscopic blood examinations on D15 or D30 and blood/heart qPCR studies performed on animals sacrificed after CP-treatment (see the results section); parenthesis in the congenital transmission rate column indicate the estimated theoretical maximum rate of congenital infection according to the numbers of studied pups per mouse group.

The 8 congenitally-infected cases were detected before CP-induced immune suppressive treatment, by microscopic blood examination on D15 (for 3/6 TcII congenital cases) or D30 (for 3/6 TcII and 2/2 TcVI cases). IgG1 and IgG2a antibodies have been detected in the 4 surviving congenitally-infected pups (2 TcII and 2 TcVI; see below and Table 3 for mortality rates; antibodies have been synthetized by pups since the latter have been suckled by uninfected mice), confirming congenital infection. Antibody investigations and qPCR studies (on the blood and the heart) in the other 517 examined pups, performed before and after CP-treatment, respectively, remained all negative.

**Table 3:** Mortality rate, blood and heart parasitic loads and antibody levels in congenitally infected pups.

Parasite genotype	Mortality rate n/N (%)^a^	Blood parasites^b^ p/mL (m ± SEM)	Heart parasites^c^ p/50 ng DNA m ± SEM	IgG1 Ab^d^	IgG2a Ab^d^
**TcII**	4/6 (66.7)	**D**: 4.0±2.1×10^7^	**D:** 7.4±2.6×10^4^	**D:** ND	**D:** ND
		**A:** 8.8±3.6×10^4^	**A:** 2.0±1.8×10^3^	**A:** 59±0	**A:** 55±7
**TcVI**	0/2 (0.0)	**A:** 4.0±1.0×10^6^	**A:** 1.5±0.1×10^3^	**A:** 47±2	**A:** 59±6

Ab = antibodies; D = dead; A = alive;

a = cumulative mortality at D30;

b = determined by blood microscopic examination on D15–30;

c = determined by qPCR on D15–30;

d = determined by ELISA as % between negative and positive controls at D30.

It is interesting to note that TcVI congenital cases came only from IAM females inoculated with 10^6^ parasites. These dams presented a 7-fold higher mean parasitemia at delivery than those having received only 10^5^ trypomastigotes and having not delivered infected pups (mean ± SEM × 10^5^: 18.9±0.4 vs. 2.7±0.9; Fig. 2). However, parasitemias at delivery of TcII and TcVI IAM dams having delivered congenitally infected pups were similar to those of corresponding infected mice having delivered uninfected pups (TcII: 7.5±3.9×10^5^ vs 5.9±0.2×10^5^ parasites/mL; TcVI: 1.6×10^6^ vs 1.9±0.4×10^6^ parasites/mL, respectively). Moreover, all littermates of dams having induced congenital infection were not infected. The 6 TcII-infected pups belonged to 4 different litters, each numbering 5 to 8 pups (i.e. 12.5 to 40.0% of infected pups according to the littermate), whereas both pups infected with TcVI came from the same litter counting 6 littermates (i.e. 33.3% of infected pups).

### Features and growth of congenitally infected offspring

As indicated in Table 3, 4 out of the 8 infected pups (50%; these 4 being TcII-infected pups) died between D15 and D30. Congenitally infected offspring displayed acute phase-like parasitemias (microscopy), parasitic loads in heart (qPCR) and significant IgG1 and IgG2a antibody levels (on D15–32). Interestingly, the pups who died displayed the highest parasitic loads in blood and heart.

**Figure 3:**
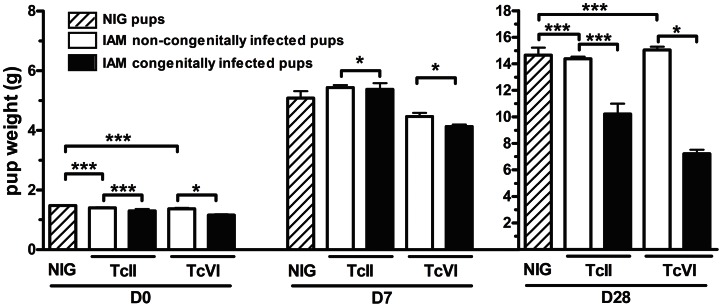
Growth of pups either uninfected or congenitally infected with TcII and TcVI. See Figure 1 for group nomenclature; uninfected offspring were born to infected or uninfected mice; * = P<0.05, ** = P<0.01, *** = P<0.001.

As shown in Fig. 3, all pups congenitally infected with TcII or TcVI presented significant lower birth weights (D0) compared to their uninfected litter mates and to the pups of NIG control animals. Moreover, despite that they were suckled by NIG females, the pups growth was strongly impaired leading to reduced weight gains of 30% (TcII) to 52% (TcVI) on D28 compared to uninfected controls.

### Search for breast-feeding transmission of *T. cruzi*

Cumulative mortality rates at the end of the breast-feeding period was of 48.2% to 90.0% for NIG pups suckled by sick IAM dams infected with TcI, TcII and TcVI (n = 50 to 112, according to the mouse group), whereas it was only of 5.3% for NIG pups suckled by TcI-infected IBM mice (n = 19). Parasitic DNA was detected neither in whole DNA extracts of 20 recently dead pups (randomized selection among TcVI pups), nor in blood and heart of alive pups (TcI-IAM: n = 17; TcI-IBM: n = 18; TcII-IAM: n = 58; TcVI-IAM: n = 30) either at D30 or after CP-immune suppression.

## Discussion

Altogether, our results obtained in mice infected with the X10 (TcI), Y (TcII) and Tulahuen (TcVI) strains show that: i) acute infection occurring after the zygote implantation time in the uterus, or close to delivery, prevents or severely jeopardize gestation outcome (inducing pup mortality and intra-uterine growth retardation); ii) gestation during chronic infection results in intra-uterine growth retardation; iii) re-inoculation of TcVI parasites during gestation in such mice strongly increases pup mortality; iv) congenital infection remains a rare consequence of infection; v) PCR is not convenient to detect congenial infection close to delivery; vi) transmission of parasites by breast milk is unlikely.

Our results clearly show that inoculation of TcI or TcII in mice some days before mating (IBM mice) allows gestation to start, but stops it promptly in most animals. Similar inhibition of reproductive capacity has been previously reported by us and others in mice infected with TcVI parasites [22], [23], [50]. This indicates that, for the three strains tested, the intense parasite multiplication associated with the early acute phase (ascending phase of parasitemia) prevents the gestation outcome when it occurs around or soon after the time of zygote implantation in the uterus. When parasite inoculation and acute infection with TcI, TcII and TcVI occur later during gestation (IAM mice), the litter sizes remain unmodified but the fetuses undergo strong growth retardation since all pups display low weights at delivery and some of them die, confirming previous observations [26]–[28]. As for the previous situation this effect is independent of the parasite strain. Interestingly, live pups from such dams are able to recover quickly to a normal growth if suckled by uninfected dams, indicating the reversibility of such an unfavorable issue. As far as we know, such effects have not been reported in human studies performed in endemic areas, probably because such situations combining an acute infection a short time before or during pregnancy are extremely rare.

Chronic infection, also for the three strains tested, induces reversible intrauterine growth retardation. This is in line with our previous results obtained in mice infected with TcVI parasites [29]. Interestingly, reinfection with TcVI in previously chronically infected animals induces a particularly high pup mortality rate. This challenging result indicates that the re-inoculated parasites are not immediately destroyed by the acquired immune response as could be expected, but are again able to multiply in inducing a new strong inflammatory response [51], detrimental to gestation outcome. Since the occurrence of pregnancy during chronic infection is the most common situation found in Latin American endemic areas, re-inoculation of parasites during gestation (in areas where vectors have not yet been eliminated) might have more severe consequences [52], suggesting that vector control (impeding subject re-inoculation) might have a secondary unexpected beneficial effect on pregnancy outcome. The contradictory reports on the frequency of abortions, stillbirths, premature births and low birth weight occurring in such women compared to the uninfected ones living in the same areas [8]–[12], might be due to inaccurate data, given the difficulties at estimating the frequency of abortions and stillbirths.

The mechanisms of such infection-associated effects on gestation outcome remain to be explored. In the first phase of gestation, they might relate non-exclusively to inhibitions of implantation (as shown previously in TcVI mouse infection when parasite inoculation occurs 7 to 10 days before mating; [44]), or interferences in the progesterone/estrogen balance necessary to maintain gestation. Indeed, *T. cruzi* is able to synthetize estrogens in the presence of steroid precursors [53], as well as to infect hormone-producing glands such as adrenals and ovary [54], [55]. In the later phase of gestation, infection induces pup mortality. The possibility of lethal congenital infection cannot be discarded. Unfortunately, it was not possible to study dead pups at delivery since collection of blood samples for microscopic examination was not feasible, and, as indicated below, PCR studies performed at birth are not convenient to confirm congenital infection. However, most likely, other mechanisms can have cause their death, as previously shown in TcVI acute infection inducing ischemic necrosis of the placenta and fetus and boosting the production of abortive cytokines (such as TNFa and IFNg), without fetal infection [22], [56], [57]. Interestingly, the resulting final harmful effect on gestation outcome has been observed in dams infected with the low virulent X10 TcI strain (inducing low parasitemias), as well as the higher virulent Y TcII or Tulahuen TcVI strains, whereas all three strains induce strong IFNg/inflammatory responses [58], [59]. Nevertheless, this does not exclude the possibility of other effects with still more highly virulent strains from the same or other genotypes.

There is no significant effect of gestation on the parasite levels observed in acute infection, whereas a limited boosting effect was observed on chronic parasitemias at least with TcII and TcVI. This is in line with previous reports in chronically infected pregnant women displaying higher parasitemia than non-pregnant ones [60]–[64]. This limited effect on chronic parasitemias might be associated with some degree of gestation-associated immune depression (imbalance of types 1 and 2 immune responses and increased amounts of Treg cells; [62], [65]), probably insufficient enough to modify significantly the high parasite levels produced in acute infection.

Congenital infections with TcII and TcVI genotypes have been detected by observing live blood parasites in 3 to 4% of live pups from acutely infected dams. Considering the numbers of studied pups (28 to 168 per group), it can be extrapolated that, if congenital infection occurs in the other studied group/genotypes, it should remain a rare phenomenon with a frequency below 1.4 to 3.6%. However, these frequencies might have been underestimated if some dead pups would have been also congenitally infected (see above). As far as we know, this is the first study exploring congenital infection using CP-induced immune suppression associated to parasitological and qPCR detection methods, in order to seek cryptic infection. Cryptic congenital infection has been detected in none of the other examined pups. This is in line with most previous reports having investigated congenital transmission in experimental acute infections by using parasitological procedures [23], [25], [26], [30], [32]–[34], but contradicts more recent studies having only used PCR in pups without having verified the occurrence of an actual infection ([38], [39]; see below). Congenital infection has been detected neither in TcI nor in chronic TcII or TcVI infections, in which dam parasitemias were particularly low. This suggests that parasite virulence would be a necessary factor to get a significant maternal threshold parasitemia to cope with the endogenous placental defenses, and finally to successfully encounter an optimal route of transmission [3]. However, if virulence seems necessary to drive such congenital transmission, it is likely not sufficient enough. Indeed, TcII and TcVI IAM dams have delivered congenitally infected as well as uninfected pups and displayed parasitemias similar to those of infected mice having delivered only uninfected litters. This highlights that other unknown factors that might be strain/genotype-dependent, such the capacity to multiply in phagocytic and trophoblastic cells [66]–[68], have to be associated to deliver congenitally infected offspring. Our data also raise the question of the suitability of the mouse model for studying *T. cruzi* congenital infection, since the maternal-fetal transmission rates reported in human congenital cases are higher than those presently observed in mice (raising 53% in the rare cases of acute infection and averaging 4–5% in the majority of pregnant women that are chronically infected [3]). Indeed, the differences in placentation [43] and overall the durations of gestation (38 vs 3 weeks in human and mouse, respectively), likely contribute to explain such divergences.

Another important information derived from this study is that the detection of parasitic DNA seems not convenient to confirm a congenital infection, particularly in samples collected close to birth. Indeed, parasitic DNA could be detected up to 32 to 62% of pup before D35, whereas congenital infection was not confirmed. Indeed PCR does not distinguish between the DNA of parasites living or dead and the half-life of parasitic DNA released from dead trypanosomes in an infected host remains to be studied. This agrees with previous data showing parasitic DNA persistence in completely cured mice who were treated with trypanocidal drugs [46]. Such persistence of parasitic DNA might allow its transfer from dams to fetuses and its detection in offspring after birth. The high inoculum of parasites used to inoculate mice probably induced abundant DNA fragments. Such parasite loads most certainly are not verified in chronic pregnant women. Indeed, the need for a reliable diagnosis of congenital Chagas infection is particularly relevant to the human situation in which a therapeutic decision has to be taken. PCR is presently under evaluation and has not been validated yet for the diagnosis of congenital infection, justifying the current recommendations to perform it by searching blood parasites through microhematocrit or microstrout concentration methods and analysis of IgG antibodies in infants of 8 or more months of age [69]. The results of PCR/qPCR, used as diagnosis tool of congenital infection, have therefore to be interpreted cautiously, though its use on samples taken at different times after birth might improve the detection of congenital infection [70], [71].

The follow-up of congenitally-infected pups shows higher parasitemias and parasitic loads in the heart of dying pups, emphasizing the role of virulence of parasite genotype as a relevant factor of mortality. Interestingly, similar observations have been done in human cases of congenital Chagas disease [3], [72]. Another important point to highlight is that the intrauterine growth retardation undergone by congenitally infected pups is particularly severe and their cachexia (associated with the acute infection; [73]) cannot be reversed one month after birth (despite of normalized suckling by NIG dams), compromising their long-term growth and health status.

Although our study of parasite transmission by breast-feeding (cross-fostering experiments) has not been extensive, no elements in our experimental results favor such a possibility, which confirms previous reports in mice [74], [75] or rats [34], [76]. The higher mortality rates observed in the NIG pups suckled by IAM dams, compared to the NIG pups nursed by IBM dams, could be related to the severe sickness of IAM mice being in full acute phase at the time of suckling. By contrast, the IBM dams, infected earlier, had already recovered from their acute phase, and likely were able to provide a better quality breast-feeding.

Our study comparing three *T. cruzi* strains belonging to three different genotypes indicates that mainly acute infection is jeopardizing mouse gestation outcome, whereas congenital infection remains a rather rare consequence of dam infection. Further studies using various strains and other parasite genotypes are needed to explore the mechanisms of such effects on gestation and to appreciate the role of virulence, and others parasitic factors, conditioning the establishment of a congenital infection.
